# The Innate Immune Cell Profile of the Cornea Predicts the Onset of Ocular Surface Inflammatory Disorders

**DOI:** 10.3390/jcm8122110

**Published:** 2019-12-02

**Authors:** Amaya Pérez del Palomar, Alberto Montolío, José Cegoñino, Sandeep Kumar Dhanda, Chit Tong Lio, Tanima Bose

**Affiliations:** 1 Mechanical Engineering Department, University of Zaragoza, 50009 Zaragoza, Spain; 2 Biomaterials Group, Aragon Institute of Engineering Research, University of Zaragoza, 50009 Zaragoza, Spain; 3 La Jolla Institute for Immunology, La Jolla, CA 92037, USA; 4 Chair of Experimental Bioinformatics, Technical University of Munich (TUM), 85354 Freising-Weihenstephan, Germany; 5 Institute for Clinical Neuroimmunology, Ludwig Maximilian University of Munich (LMU), 82152 München, Germany

**Keywords:** ocular surface disease, autoimmune diseases, dendritic cells, inflammation, conjunctiva, lymphocytes

## Abstract

Ocular surface inflammatory disorder (OSID) is a spectrum of disorders that have features of several etiologies whilst displaying similar phenotypic signs of ocular inflammation. They are complicated disorders with underlying mechanisms related to several autoimmune disorders, such as rheumatoid arthritis (RA), Sjögren’s syndrome, and systemic lupus erythematosus (SLE). Current literature shows the involvement of both innate and adaptive arms of the immune system in ocular surface inflammation. The ocular surface contains distinct components of the immune system in the conjunctiva and the cornea. The normal conjunctiva epithelium and sub-epithelial stroma contains resident immune cells, such as T cells, B cells (adaptive), dendritic cells, and macrophages (innate). The relative sterile environment of the cornea is achieved by the tolerogenic properties of dendritic cells in the conjunctiva, the presence of regulatory lymphocytes, and the existence of soluble immunosuppressive factors, such as the transforming growth factor (TGF)-β and macrophage migration inhibitory factors. With the presence of both innate and adaptive immune system components, it is intriguing to investigate the most important leukocyte population in the ocular surface, which is involved in immune surveillance. Our meta-analysis investigates into this with a focus on both infectious (contact lens wear, corneal graft rejection, Cytomegalovirus, keratitis, scleritis, ocular surgery) and non-infectious (dry eye disease, glaucoma, graft-vs-host disease, Sjögren’s syndrome) situations. We have found the predominance of dendritic cells in ocular surface diseases, along with the Th-related cytokines. Our goal is to improve the knowledge of immune cells in OSID and to open new dimensions in the field. The purpose of this study is not to limit ourselves in the ocular system, but to investigate the importance of dendritic cells in the disorders of other mucosal organs (e.g., lungs, gut, uterus). Holistically, we want to investigate if this is a common trend in the initiation of any disease related to the mucosal organs and find a unified therapeutic approach. In addition, we want to show the power of computational approaches to foster a collaboration between computational and biological science.

## 1. Introduction

The ocular surface, which comprises of the cornea, limbus conjunctiva, and tear film, plays a key role in the visual system. Its integrity is crucial for the health and normal function of the eye, and as a result it is equipped with a delicate mucosal immune system to help prevent or limit damage. It is comprised of both the innate and adaptive immune system, and the prevention of ocular surface diseases depends on the appropriate activation and regulation of these inter-connected branches of the immune system, which in turn rely on various molecular sensors and receptors. This wide spectrum of diseases can occur as a result of pure infection, stemming from “external’” factors, such as contact lens injury, or ‘internal’ factors, including autoimmune diseases such as rheumatoid arthritis and Sjögren’s syndrome [1,2]. Ocular surface diseases represent a significant unmet medical need and are a substantial burden to patients, families, and healthcare systems. In the United States, annual medical costs per patient with only dry eye are estimated to be $783 per year, with overall cost calculated to be approximately $3.84 billion per year. In addition, societal costs encompassing the loss of productivity are estimated at $11,302 per year, with the overall cost calculated to reach $55.4 billion per year [3]. Since there are only very limited numbers of effective and generalized treatments for this group of diseases, more investigation into its etiology and underlying mechanisms is crucial [4,5,6].

The innate nature of the immune cells (i.e., dendritic cells, macrophages, or natural killer cells) includes recognition of the wide variety of danger signals during infection, inflammation, and tissue injury. Their adaptive component involves antigen-experienced memory-like cell characteristics, which includes the ability to produce cytokines, cytolytic molecules, and growth factors after insult from infection. Among the innate immune cell types, dendritic cells (DCs) are an important player, forming an essential interface between the innate sensing of pathogens and the activation of the adaptive immune response [7]. DCs, if not γδ and resident memory T cells, are quite an important population in bridging the activation between the adaptive and innate immune system pathway.

It is inspiringly shown that there is an 80-fold increase in the number of myeloid DCs in the airway mucosa layer and in bronchoalveolar lavage fluid in a mouse or rat model of asthma. There are two pathways to control the inhalational tolerance: The first silences antigen (Ag)-reactive T cells, along with an induction of expanding regulatory T cells in the lymph nodes. They ingest inhaled Ags and migrate to the draining lymph nodes with an upregulation of the homing receptor CCR7, and DCs activate T cells in the draining lymph nodes to induce tolerance. On the other hand, plasmacytoid DCs (pDCs) directly suppress the capacity of myeloid DCs to generate effector T cells and they can also stimulate the generation of Treg cells, possibly in an inducible T-cell co-stimulator ligand (ICOS-L)- dependent manner. Following short-term allergen inhalation, there is fast recruitment of myeloid DC precursors from the circulation to the airway mediated by a chemoattractant receptor signal relay initiated by CCR2, followed by CCR7, to complete the journey of DCs to the mesenteric lymph node for Th2 priming [7]. On the other hand, Langerhans cells (LCs), discovered by Paul Langerhans in the mid-19^th^ century, functioning as skin-resident antigen presenting cells (APCs) was only recently appreciated [8]. Recently, LCs have shown to be important in the functions of the mucosal epithelial layer lining the ocular, vaginal, cervical, and oral surfaces [9,10,11]. LCs have been important in initiating the adaptive immune responses by presenting the antigens to the naïve T cells [12]. Similar to the asthma model, the DCs have been shown to play a significant role in the progression or control of autoimmune diseases. The mere change in the number of DCs though cannot be directly related to autoimmunity, alternation in their functionality might indicate autoimmune infestation [13], pDC-mediated production of type I interferon has been suggested as a common pathway that leads to pathogenesis in psoriasis, type I diabetes, and systemic lupus erythematosus (SLE).

Similar to our previous observation with the homogeneous presence of immune cell types in barrier tissues with a resident memory cell function, our current meta-analysis also attempts to find the quantitative traits of dendritic cells, especially LCs, in the mucosa of the ocular surface, like other mucosal organs (skin, lung, intestine, cervix, gastric mucosa) [6]. The ocular surface immune system has been investigated to have different immune cell components, both in the innate and adaptive branches and the phenotype, though the functions of those components are still under-investigated. We have selected a spectrum of ocular surface diseases in our study with underlying etiologies, both in the branches of infection and autoimmune diseases. The selected diseases in our study are dry eye, glaucoma, Graft-vs-host disease (GvHD), keratitis, scleritis, Sjögren’s syndrome, and contact lens wear. We have mostly found the involvement of dendritic cells among our chosen studies, followed by cytokine IL6, IL17, a prominent helper (CD4^+^, Th), and cytotoxic (CD8^+^, Tc) cells. Along with the predominant immune population contributing to the manifestation of the disease spectrum, we have conglomerated the innovative diagnostic methods. This paper has the vision of opening several new opportunities relating to the anti-inflammatory paradigm of treatments in ocular surface inflammatory disorders (OSIDs).

## 2. Method

### 2.1. Study Retrieval and Selection

**Figure 1:**
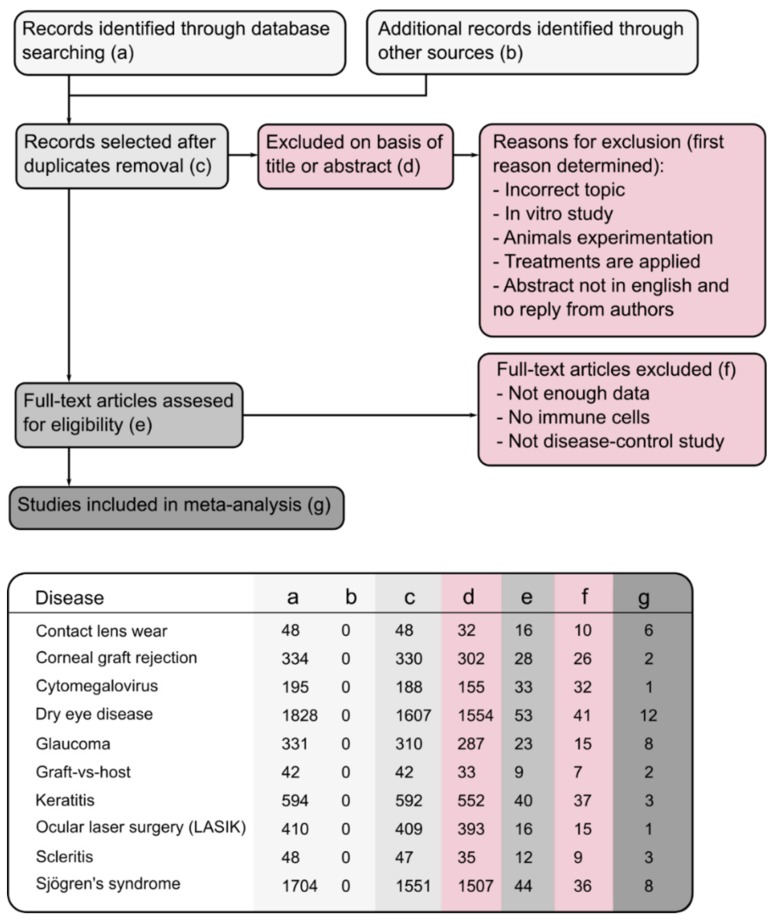
Flowchart of study selection process. The flowchart was initiated with 5534 studies extracted through several databases. After a rigorous evaluation, 46 studies were selected which matched with the inclusion criteria. (**a–g**) in the lower panel describes the way we screened the studies. (**f**) represents the excluded studies whereas (**g**) represents the number of studies used for the further analysis.

We followed the meta-analysis of observational studies in epidemiology guidelines for reporting systematic reviews and meta-analysis for this paper [14], summarized the exact procedure in Figure 1, and searched the following databases systematically: Medline, PubMed, Embase, Google Scholar, and Web of Science (citation index) from 1990 to 2019, and this search was performed from April to May 2019. For our literature research, we included a combination of keywords, such as the specific disease (corneal lenses wear, corneal graft rejection, cytomegalovirus, dry eye disease, glaucoma, Graft-versus host disease (GvHD), keratitis, ocular laser surgery (LASIK), scleritis, and Sjögren’s syndrome), different kinds of immune cells (B lymphocytes OR Plasma Cells OR T lymphocytes OR CD8^+^ T cells OR CD4^+^ T cells OR Th1 cells OR Th2 cells OR Th17 cells OR Treg cells OR memory T cells OR Neutrophils OR macrophages OR γδ-T cells OR Natural killer T cells OR dendritic cells OR innate lymphoid cells), and terms related to ocular surface (eye OR ocular surface OR ocular). The readers can access the full details of all search strategies and search terms used in the manuscript from us on request. Two authors (A.M. and J.C.) completed the literature search independently. Furthermore, these authors further cross-checked the reference lists of all selected articles to include other relevant studies.

We performed the exclusion criteria in two steps. At first, matching was performed only to the title or abstract. We mainly excluded articles if they discussed an incorrect topic, an *in vitro* study, animal studies, or treatments applied. Figure 1 displays the excluded papers by column (d). We then fully assessed the remaining papers, and the full-text articles were excluded if not enough data were presented, if no immune cells were quantified, or if they did not include a comparison between patients and controls. Figure 1 shows the excluded papers in this second step in column (f). The final studies obtained after the systematic review are represented column (g). The lower panel of Figure 1 represents the number of studies filtered in each of these steps (a–g) for the selected diseases. There was one paper for the cytomegalovirus and another for ocular laser surgery (LASIK) that did not satisfy our criteria. Therefore, these diseases were removed from our meta-analysis. For the Supplementary Figures S1–S8, each one is constructed in the same way: The specific papers used are detailed in the first columns and the presence of different cells (dendritic cells, IL6, CD25, CD3), both for controls and patients, are depicted. Both the mean and standard deviation are shown for the total number of control and disease samples. The weight column is the relative importance of each study, regarding the number of samples. Finally, the last two columns reflect the increase (right side of the graph) or decrease (left side of the graph) of those specific cells in patients related to the controls.

### 2.2. Statistical Analysis

We analyzed the data using RevMan software version 5.3 (Review Manager; Copenhagen: The Nordic Cochrane Centre, The Cochrane Collaboration, 2014). For clusters of articles for each of the diseases, meta-analysis was performed. Data entered in RevMan included these parameters: mean and standard deviation value of the concentration of different immune cells at ocular surface for patients and controls and the corresponding number of participants in each group. For the statistical method, we chose inverse variance (IV). In this procedure, the mean and standard deviations are entered directly into the software. In the inverse variance method, weight given to each study is the inverse of the variance of the effect estimate. This choice of weight minimizes the uncertainty of the pooled effect estimate. For pooled data, the I^2^ statistic was used to estimate heterogeneity. I^2^ values of 50% or more were considered to indicate substantial heterogeneity, and the random-effects model was then used; in other cases, the fixed-effects model was used. Finally, the chosen method selected for the effect measure was the standardized mean difference (SMD) with its 95% confidence interval (CI). Positive SMD represents a higher concentration of immune cells in patients than in controls.

## 3. Results

### 3.1. Dendritic Cells are the Major Immune Cells Responsible for Ocular Surface Diseases 

**Figure 2:**
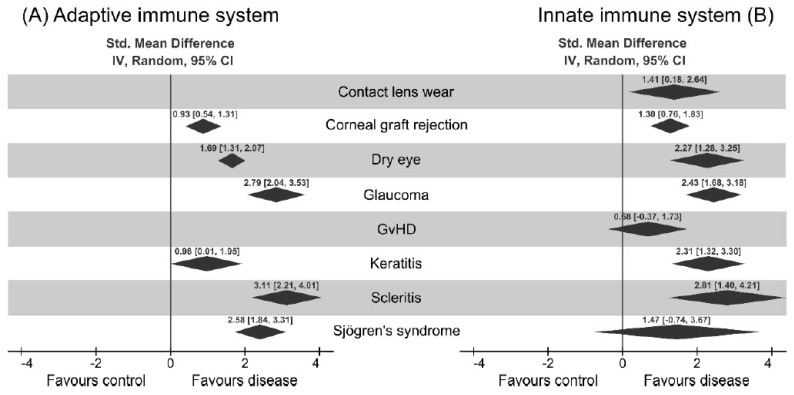
Analysis of different immune cell components in the eight chosen ocular surface diseases. The global standard mean difference for each disease obtained by our proposed meta-analysis is shown as a part of adaptive immune system (**A**) and innate immune system (**B**). The diamonds represent the standard mean difference and the 95% confidence interval for each disease. It can be seen that the presence of immune cells is higher for every analyzed disease. A detailed description of each of the diseases and the different cells involved can be found in the Supplementary Material (Supplementary Figures S1–S8).

We divided the immune cell components into two major sections depending on their predominant functions. The major cells associated with the adaptive immune system are T- (Th, T_C_, T_RM_) and the sub-division of Th cells, such as Th17, Tregs, and the B-lymphocytes. The major immune cells associated with the innate immune system are dendritic cells, platelets, neutrophils, monocytes, and macrophages. The raw data for each of the diseases to perform individual cell type analysis for adaptive and innate immune branches is provided in the Supplementary Figures S1–S8. A representative example of the overlay of the innate and adaptive immune system is shown in Figure 2. Along with the immune cells, we also retrieved the importance of cytokines associated with both of the sub-divisions of the immune system. Among the several cells and inflammatory mediators that are important for the ocular surface diseases, we found the number of research papers following this decreasing order: dendritic cells (19) > IL6 (15) > IL17 (8) > CD4^+^ and CD8^+^ T cells together (7). The eight diseases that matched the inclusion criteria of the study mentioned in the method section are: contact lens wear [15,16,17,18,19,20], corneal graft rejection [21,22,23], dry eye [6,24,25,26,27,28,29,30,31,32,33,34], glaucoma [35,36,37,38,39,40,41,42], Graft-vs-host (GvHD) [43,44], keratitis [45,46,47], scleritis [48,49,50], and Sjögren’s syndrome [28,29,32,33,34,51,52,53]. This is a combination of different ocular surface disorders, the range of whose etiologies lie from the infectious encounter to autoimmune factors. Among these diseases, we could not find any involvement of adaptive immune system factors in case of contact lens wear and GvHD. Regarding the contact lens wear and GvHD (Supplementary Figures S1 and S5), we have found the significance of dendritic cells only (langerhans cells for the contact lens wear). For the corneal graft rejection, combinations of innate (monocytes, macrophages) and adaptive (CD3, CD25) factors are responsible (Supplementary Figure S2). For the dry eye disease, we found the maximum number of innate and adaptive factors responsible for initiating the dendritic cells, the platelets to a series of cytokines (IL17, IL23, IL33, IL4, IL5, IL6, IL10, IFNγ, transforming growth factor (TGF)-β), and the chemokines (CCL2, CXCL3, CXCL9, CXCR4, CXCL10, CXCL11, CXCL12) in the adaptive component (Supplementary Figure S3). Dry eye disease is a multi-factorial disease, in which the clinical characteristics can be either systematic (SLE, rheumatoid arthritis (RA), hypothyroidism), or idiopathic, and sometimes requiring ocular surgery. This was properly displayed through the huge number of immune factors responsible for dry eye disease in comparison to other diseases. For the studies of glaucoma, we also found many factors common to the dry eye disease, along with the plasma cells in the adaptive part (Supplementary Figure S4). For the infection-related keratitis, we considered the papers related to the fungal, bacterial, and stromal keratitis. Among the different components, the intriguing papers were those involved with CD19, CD20, anti-inflammatory component IL10, and pro-inflammatory component IL1β (Supplementary Figure S6). For scleritis, the one component which stands out, both in the innate and adaptive component, is the myeloid cell markers (Supplementary Figure S7). For Sjögren’s syndrome, the adaptive components play major roles in comparison to the innate factors. The one adaptive component most mentioned is IL17 (Supplementary Figure S8).

### 3.2. Upregulation of Cytokines Mediating Th1, Th17, and Treg Activation 

**Figure 3:**
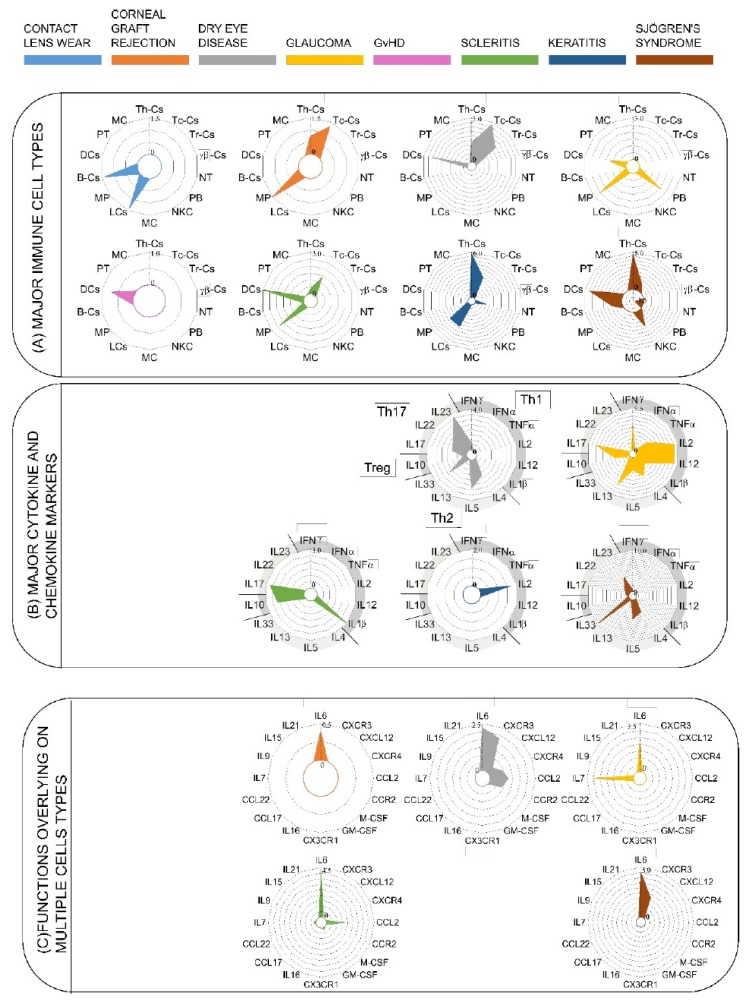
Radar plots to explain the relationships of the ocular surface diseases and the immune cells and their mediators viz cytokines and chemokines. (**A**) Major immune cells, (**B**) major cytokine and chemokine markers, (**C**) functions overlying on multiple cell types. Each color represents a different disease; these are defined at the top of the figure. Empty spaces correspond to empty radar plots for that disease. The different scales for each radar plot correspond to the standard mean difference between controls and patients calculated in the forest plots (see supplementary material for extended data, Supplementary Figures S1–S8, Table S1) for each cell. Each circle represents an increase of 0.5 in the standard mean difference. The following abbreviations have been defined: T helper cells (Th-Cs); T cytotoxic cells (Tc-Cs); T resident cells (Tr-Cs); γδ-Cs (γδ T-Cs); neutrophils (NT); plasmablasts (PB); natural killer cells (NKCs); mast cells (MCs); Langerhans cells (LCs); macrophages (MPs); B cells (B-Cs); dendritic cells (DCs); platelets (PT); monocytes (MCs).

**Figure 4:**
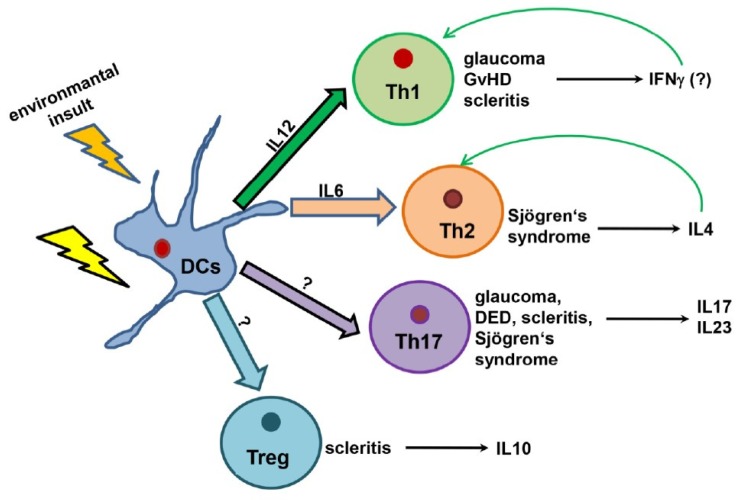
Possible interaction of dendritic cells and other components of T cell differentiation in the ocular surface. A dendritic cell is the key component in ocular surface diseases. Following the environmental insult, dendritic cells try to eradicate the pathogen, if not eradicate the different T cell differentiated pathways that are activated to fight with the pathogens.

This meta-analysis reveals the interesting patterns of immune cell composition among the ocular diseases in our study (Figure 3A). Notably, the exclusive role of innate immunity, mediated through the dendritic cells, is observed only in contact lens wear. It is also interesting to note that the dendritic cells were studied across different ocular diseases, except for Scleritis and corneal graft rejection. Dendritic cells produce the signals for the activation of different types of T cells. Discovered by Steinman and Cohn in 1974, dendritic cells act as a converter station, which recognizes the input signals coming from the pathogens and interprets the signals followed by the processing and conversion to the output signals, which influences the induction of distinct Th cell subtypes. Three common output signals known from the dendritic cells are antigen presentation, co-stimulatory molecule expression, and a direct contribution by the DC to the cytokine milieu for ensuring the specific Th-subset. In other words, DCs act as sentinels for the uptake of antigens, upregulate CCR7, and migrate to the draining lymph nodes, where the interaction of T cells occurs regularly. The mucosal immune system interacts with the pathogens more ubiquitously, and thus the role of dendritic cells in those organs is quite prominent in mediating the role of APCs to the naïve T cells. For example, DCs in Peyer’s patches have been shown to express IL10 and IL4, in contrast to the similar cells in peripheral lymph nodes, which express IFNγ and IL12. In our analysis, we have observed that dendritic cells act as a core of network signals, activating several network pathways. As clearly shown in Figure 3B, the radar plots for different diseases are upregulated, such as Th17 (IL17, Il22, IL23) and Th2 (Il33, IL4, IL5), cytokine markers for dry eye diseases, (Th1 (IFNγ, IL2, IL12), Th2 (Il4, Il5, IL13), Th17 (IL17), Treg (IL10)) cytokines for glaucoma (Th17 (Il17), Treg (IL10), Th1 (IL1β)) scleritis (Th1 (IL2)) GvHD (Th2 (IL33, IL4, IL5), Th17 (IL22, IL23)), and Sjögren’s syndrome. Thus, it can be postulated from Figure 4 that DCs act as an initiator of the activities to modulate the different types of T_H_ cells. From the literature, it is known that there are other APCs as well to activate the T cells, such as macrophages and B-cells. Additionally, DCs in action are more potent (100 times more potent than macrophages) [54]. 

We further investigated the role of cytokines and chemokines in shaping the immune response in ocular diseases. We took into consideration the characteristic cytokines for each of the cell types, and we separated those cytokines according to their association with different differentiated Th cytokines, as depicted in the radar plot B: IFNα, IFNγ, TNFα, IL2, IL12, IL1β (Th1); IL4, IL5, IL13 (Th2), IL10 (Treg), IL17, IL22, IL23 (Th17). As shown in the analysis, there is a major association with Th1-cytokines in the case of glaucoma only (IL2, IL12), and a minor association with keratitis (IL2) and scleritis (IL1β). On other hand, Th2-associated cytokines are only important in the case of dry eye disease (IL4, IL5, IL33) and Sjögren’s syndrome (IL33). Furthermore, Treg-associated cytokines can hardly be found in those selected diseases, whereas Th17-associated cytokines (IL23, IL17) can be found in all the diseases except Keratitis. The diseases that associate with the differentiated Th cells are dry eye disease, glaucoma, scleritis, keratitis, and Sjögren’s syndrome. The association with the number of diseases is strongest in the case of Th17 (Figure 3B). For Figure 3C, we took into consideration the molecules that have got overlapping functions in both innate and adaptive immune systems. The classical example of this is IL6, which has got both pro- and anti-inflammatory properties [55]. This was seen in most of the diseases (corneal graft rejection, dry eye disease, glaucoma, scleritis, Sjögren’s syndrome) in our study. The other examples are CCL2, IL7, CXCR3, and CXCL12, which are found in scleritis, dry eye disease, glaucoma, dry eye disease, and Sjögren’s syndrome. We further extend this meta-analysis in a possible hypothesis (summarized in Figure 4 of dendritic cell interactions with T cells, which is based on the consideration of cytokine milieu observed in this context. The dendritic cells respond to environmental insults through the activation of different T cell subsets, which depends on the cytokine milieu manifested in the disease. Diseases like glaucoma, GvHD, and scleritis biases the immune response to IFNγ release through IL12-mediated Th1 activation, whereas Sjögren’s syndrome activates Th2 immunity. The activating signal for Tregs and Th17 need to be investigated further. If we try to imagine the picture from our analysis and existing literatures, DCs are able to fight with the pathogens in the case of contact lens wear, corneal graft rejection, and GvHD. The exact molecular mechanism for the action of DCs is still lacking in the literature. In the case of other diseases, there is a need for the recruitment of T cells and more specialized action of an adaptive immune system. According to current understanding, dendritic cells activate Th1 cells through IL12, and activated Th1 secretes IFNγ, which in return re-activates the Th1 cell. In the case of Th2, the T cell activation occurs through IL6, and in this case activated Th_H_2 cells secrete IL4, which reactivates the Th2 cells. We speculate that Th1 and Th2 activation can happen in the case of glaucoma, GvHD, scleritis, and Sjögren’s syndrome. The mechanism for Treg and Th17 is still not quite clear, though many ocular diseases take the pro-inflammatory path of Th17. What we now know is that the Th17 cells secrete IL17, IL23 in the case of glaucoma, dry eye disease, scleritis, and Sjögren’s syndrome, and the anti-inflammatory pathway of Treg is taken by the cells in the case of scleritis.

### 3.3. Clinical Factors Associated with Ocular Surface Diseases

**Figure 5:**
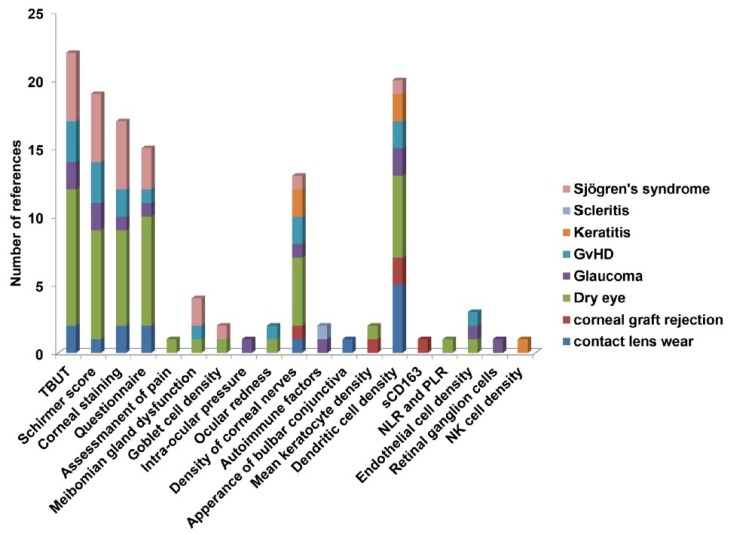
Illustration of importance of different clinical tests in ocular surface disorders. 19 clinical factors were correlated with eight different ocular surface diseases. The distribution was shown among the selected papers for the analysis. Graft-vs-host disease (GvHD); tear break-up time (TBUT); platelet-lymphocyte ratio (PLR); neutrophil-lymphocyte ratio (NLR).

The most common clinical tests associated with ocular surface diseases found in our study are tear break-up time (TBUT), tear secretion by the Schirmer’s score, corneal fluorescein staining to test the corneal opacity, meibomian gland dysfunction, and a questionnaire to assess the symptoms as analyzed from the literatures selected in the method section. The other tests that were less common in the OSIDs, but were still used for some of the diseases, are testing of intra-ocular pressure, ocular redness (hyperemia), density of corneal nerves, autoimmune factors, sCD163 and appearance of bulbar conjunctiva (thickness and cell density), assessment of pain, mean keratocyte density, Langerhans cell density, endothelial cell density, number of retinal ganglion cells, and natural killer (NK) cell density. Figure 5 illustrates the propensity of all of the clinical tests in our eight selected ocular surface diseases. Other than the usual clinical tests, the two tests that stand out in Figure 5 are the dendritic cell density and the density of corneal nerves. Some of the clinical factors that are specific for different ocular surface diseases are the assessment of pain, the neutrophil-lymphocyte ratio (NLR), and the platelet-lymphocyte ratio (PLR) in dry eyes, intra-ocular pressure, retinal ganglion cells in glaucoma, the appearance of bulbar conjunctiva in contact lens wear, soluble marker sCD163 in corneal graft rejection, and natural killer cell density in keratitis. Altogether, the assessment of dendritic cell density can be a new feature in determining ocular surface diseases.

## 4. Discussion

Ocular surface disorder is a complicated disorder in the sense that there might be an overlap between different diseases, and the etiologies of the diseases can also be extremely variable. We can take an example of dry eye and Sjögren’s syndrome to explain the complexity. In most of the references studied, DED patients were divided into two groups—the Sjögren’s syndrome group and the non-Sjögren’s syndrome group. The clinical features checked for both the diseases are dry eye symptoms (>3 months), high OSDI scores (>13 points), abnormal TBUT (<10 s), low Schirmer I test (<10 mm), and positive corneal fluorescein staining. However, the distinction between these two diseases is not quite clear, but the researchers are continuously making different innovative efforts to diagnose the different forms. For example, the American–European consensus group in the year of 2002 revised this classification and included oral symptoms, salivary gland involvement, serum autoantibodies, rheumatoid factors, and anti-double-stranded DNA (dsDNA). Along with the difficulty of diagnosis, multiple etiologies can be involved in dry eye diseases or Sjögren’s syndrome-like diseases, including autoimmune diseases, ageing, medications, refractive surgery, habits, diet, vitamin D, and environmental factors [24,26,28]. What can be reflected from this example is that it is not very easy to classify ocular surface diseases, but they can be represented as a spectrum in which they reflect a range of phenotypic features from mild to severe. 

DCs are central to the immune memory activation and tolerance induction. DC development takes place in the bone marrow and has tissue-residency and migratory characteristics, predominantly due to its requirement to replenish the mature DCs in peripheral tissues [55,56,57]. DCs can be classified into four broad subsets, according to the phenotype, function, and developmental origin, and they each play distinct roles in immune responses. DC classification includes plasmacytoid DCs (pDCs), tissue-resident and migrators conventional DCs (cDCs), Langehans cells (LCs), and monocyte-derived DCs (mDCs). This classification of DCs is consistent between mice and humans. There is a functional and structural difference between the tissue-resident and migratory DCs. Tissue-resident DCs express different receptors for microbe-associated molecular patterns (MAMPs) and damage-associated molecular patterns (DAMPs), and these are activated by the infections and promote the production of pro- or anti-inflammatory cytokines. After capturing different antigens, DCs migrate through the lymphatics to reach the secondary lymphoid organs, where DCs present processed antigenic peptides to stimulate the naïve T cells. The tissue-resident DCs exist in several forms with several markers in different organs, according to previous literature. Various organs where DCs display resident immune cell functions are the skin, blood, lungs, liver, gastrointestinal (GI)-tract, kidney, spleen, and lymph node. Tissue-resident DCs contribute to the prognosis of a range of diseases, such as allergies, auto-immunities, inflammation, and cancer progression [58,59,60,61,62]. It is not only the tissue-resident population but the migratory DCs as well that contribute hugely to the overall functionality of any organ [18]. For example, in mice kidneys, the DCs are replaced within 2–4 weeks after lethal irradiation. In contrast, the replacement of migratory DCs is much faster (within 7–13 days) in the vagina, airway epithelia, and gut. In the steady-state, DC progenitors migrate form the bone marrow through the blood to the peripheral tissues, where they are designated to raise distinct subsets of myeloid DCs after a final differentiation with the exception of Langerhans cells, which resided in the epidermis of skin independent of its circulating precursors. The human and mice DCs have been investigated intensively in previous literatures, but our article is mainly focused on the human experiments, except for a few examples of the animal models [58,63,64,65]. 

In our current study, we made an effort to characterize the ocular surface diseases with a common mechanism. Dendritic cells are the cornerstone of those diseases and can activate the differentiated T cells in different ways, mediating through many chemokines and cytokines. An easier explanation of this would be to activate the Th1 differentiated pathway with the mediator of IL12, which in turn secretes IFNγ, which keeps the production of Th1 cells in a negative feedback loop. Same mechanisms can be seen in the case of Th2 cells, but feedback, with the help of IL6 and IL4, is formed. In the case of Th17 and Treg pathways, these are controlled by IL17, IL23, and IL10 pathways. We also have seen the difference in the mechanism in different ocular diseases, except the contact lens wear, which is an entirely innate immune system phenomenon, involving only the Langerhans cells. In the case of glaucoma, GvHD, and scleritis, we have seen the factors regulating the Th1 pathway, whereas Sjögren’s syndrome is regulated by the Th2 pathway. On the other hand, there is involvement of Th17 pathways in the case of glaucoma, DED, scleritis, and Sjögren’s syndrome and the Treg pathway in the case of scleritis [7,66,67,68,69,70]. The reliability of these common mechanisms in different diseases can be compared with the mouse studies, which are beyond the capacity of this manuscript. Overall, this manuscript represents a combination of different factors through which ocular surface disorders can be prevented with the help of several cells and mediators, the key of which is in the activity of dendritic cells, predominantly the Langerhans type. The research in the field of Langerhans cells is overlooked in the case of skin immunity, the reason being that the deletion of Langerhans cells does not result in fatal susceptibility to a skin infection nor due to an overt autoimmunity due to lack of immune regulation. The eye, being the mucosal organ similar to the skin, also represents the importance of Langerhans cells in maintaining and modulating the tissue environment in shaping the effector immunity [71]. More understanding in the field of Langerhans cells will develop new possibilities for therapeutic approaches in the field of ocular surface diseases.

### Pitfalls and Future Perspectives

This study is a conglomeration of the observations from the publications of the last 20 years, but this is already a hint to the research direction in the field of ocular surface disorders. The number of references we have worked with did not represent a huge number—this is one of the limitations of the study. In addition, we reflected the phenotypic features of ocular surface diseases with the help of eight selected diseases. In spite of these two limitations, this study gives an indication as to which immune cells or which immune mediator can able us to alleviate the ocular surface diseases. Future studies with the knockout models with the deletion of genes for the important cytokine and chemokine factors, such as IL2 and IL12, will give new insight into the field of research.

## Supplementary Materials

The following are available online at https://www.mdpi.com/2077-0383/8/12/2110/s1, Figure S1: Meta-Analysis results for contact lens wearers. Only innate immune system is shown, because no adaptive immune cells were found, Figure S2: Meta-Analysis results for corneal graft rejection, Figure S3: Meta-analysis for Dry Eye Disease, Figure S4: Meta-analysis for Glaucoma, Figure S5: Meta-analysis for Graft vs Host Disease. Only innate immune system is shown, because no adaptive immune cells were found, Figure S6: Meta-analysis for Keratitis, Figure S7: Meta-analysis for Scleritis, Figure S8: Meta-analysis for Sjögren’s Syndrome, Table S1: Keywords used to filter the records identified through database searching in order to obtain the final studies to perform the meta-analysis.
