# Supplementary material for: The Innate Immune Cell Profile of the Cornea Predicts the Onset of Ocular Surface Inflammatory Disorders
Source: J Clin Med. 2019 Dec 2;8(12):2110. doi: 10.3390/jcm8122110 (PMC6947418; doi:10.3390/jcm8122110)
Supplement: Supplementary file 1 [file jcm-08-02110-s001.pdf]

Supplementary Material

DISEASE	KEYWORDS TO INCLUDE	KEYWORDS TO EXCLUDE
**Contact Lens Wearer**	Contact Lens, Cells, Contact Lens Wear, Patient, ramdomly	Review, *in vitro* , animal, treatment
**Corneal Graft Rejection**	Cells, T cells, CD4, corneal graft, macrophages, CD8, corneal graft rejection, lymphocytes, dendritric cells, patient, controls, neutrophils, ocular surface, conjunctival, Treg cells, Th1 cells, Th17 cells, healthy controls, memory, Th2 cells, lacrimal	Review, *in vitro* , animal, treatment
**Dry Eye**	T cells, dry, dry eye, CD4+ T cells, Plasma cells, dendritic cells, T lymphocytes, Th1, macrophages, B lymphocytes, Neutrophils, DED, Th17 cells, CD8+ T cells, Th1 cells, Treg cells, Th2 cells, memory T cells, natural killer T cells, Innate lymphoid cells, gd T cells	Review, syndrome, Sjögren, SS, animal, treatment
**Glaucoma**	Glaucoma, cells, patients, macrophages, ocular, plasma, conjuctival, lymphocites, surface, T cells, Neutrophils, dendritic cells, CD4, CD8, Treg cells, Th2 cells, Th1 cells, lacrimal, autoinmune, Th17 cells, memory	Review, blood, animals, treatment
**GvHD**	Graft-versus-Host, cells, patients, T cells, eye, CD4, CD8, Lymphocytes, lacrimal, dendritic cells, ocular surface macrophages, plasma cells, neutrophils, Th17 cells, Treg cells, Th1 cells, Th2 cells	Review, treatment, skin, bone, animal, blood, cancer
**Scletitis**	Scleritis, cells, lymphocytes, dendritic, neutrophils	Review, blood, animal, treatment
**Keratits**	keratitis, cells, neutrophils, CD8, lymphocytes, ocular surface, dendritic cells, Th1 cells, memory, Th17 cells, lacrimal, Treg cells, Th2	Review, blood, animal, treatment
**Sjögren's syndrome**	Syndrome, Sjögren's syndrome, cell, patient, SS, T cells, Lymphocytes, CD4, controls, plasma, lacrimal, plasma cells, CD+, surface, dendritic, Th17, macrophages, Th1, eye, memory, Th2, ocular, neutrophils, Treg, conjunctiva	Review, salivary, blood, treatment, labial, peripheral blood mononuclear, mouth, lip

Table 1S. Keywords used to filter the records identified through database searching in order to obtain the final studies to perform the meta-analysis.

Contact lens wear - Innate immune system

Study	Parameter	Contact lens wear Mean	SD	Total	Control Mean	SD	Total	Weight	Std. Mean Difference IV, Random, 95% CI
Alzahrani et al. (2016)	Dendritic cells	36	32	10	20	19	10	16.4%	0.58 [-0.32, 1.48]
Alzahrani et al. (2017)	Dendritic cells	55	7	25	27	4	23	15.5%	4.78 [3.63, 5.93]
Efron et al. (2010)	Dendritic cells	17	17	11	23	25	11	16.6%	-0.27 [-1.11, 0.57]
López-De La Rosa et al. (2018)	Dendritic cells	93.65	85.3	40	57.5	70.2	20	17.3%	0.44 [-0.10, 0.99]
Sindt et al. (2012)	Dendritic cells	64	71	53	29	23	10	17.0%	0.52 [-0.16, 1.20]
Zhivov et al. (2007)	Dendritic cells	78	25	33	34	3	43	17.2%	2.62 [2.00, 3.25]
Total (95% CI)				172			117	100.0%	1.41 [0.18, 2.64]

Heterogeneity: Tau² = 2.18; Chi² = 82.15, df = 5 (P < 0.00001); I² = 94%
Test for overall effect: Z = 2.25 (P = 0.02)

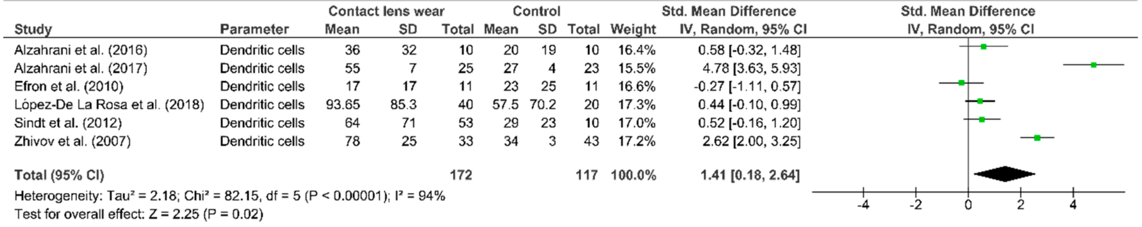

Figure 1S. Meta-Analysis results for contact lens wearers. Only innate immune system is shown, because no adaptive immune cells were found.

Corneal graft rejection - Adaptive immune system

		Corneal graft rejection			Control				Std. Mean Difference
**Study**	**Parameter**	**Mean**	**SD**	**Total**	**Mean**	**SD**	**Total**	**Weight**	**IV, Random, 95% CI**
Funding et al. (2005)	IL6	52	74	19	0.003	0.001	7	14.6%	0.79 [-0.11, 1.68]
Kuffová et al. (1999)	CD25	0.47	0.51	15	0	0.001	10	15.0%	1.14 [0.27, 2.01]
	CD3	0.67	1.17	15	0	0.001	10	15.7%	0.71 [-0.12, 1.54]
	CD4	0.37	0.61	15	0	0.001	10	15.7%	0.75 [-0.08, 1.58]
	CD8	0.87	0.83	15	0	0.001	10	14.7%	1.30 [0.41, 2.19]
	HLA-DP	2.27	1.33	15	0	0.001	10	12.8%	2.12 [1.09, 3.14]
	HLA-DR	2.8	1.33	15	0	0.001	10	11.5%	2.61 [1.49, 3.73]
**Total (95% CI)**				**109**			**67**	**100.0%**	**1.28 [0.78, 1.78]**

Heterogeneity: Tau² = 0.23; Chi² = 12.45, df = 6 (P = 0.05); I² = 52%
Test for overall effect: Z = 5.02 (P < 0.00001)

Std. Mean Difference
IV, Random, 95% CI

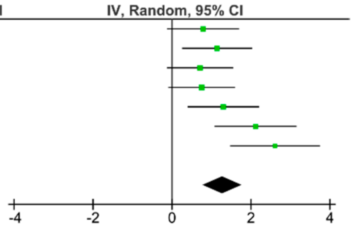

Corneal graft rejection - Innate immune system

		Corneal graft rejection			Control				Std. Mean Difference
**Study**	**Parameter**	**Mean**	**SD**	**Total**	**Mean**	**SD**	**Total**	**Weight**	**IV, Random, 95% CI**
Funding et al. (2005)	CD163	336.4	378.7	19	11.3	2.2	7	25.9%	0.96 [0.05, 1.87]
	IL6	52	74	19	0.003	0.001	7	26.5%	0.79 [-0.11, 1.68]
Kuffová et al. (1999)	CD14	2.14	1.7	15	0	0.001	10	25.2%	1.56 [0.63, 2.49]
	CD68	2	1.24	15	0	0.001	10	22.4%	2.00 [1.00, 3.00]
**Total (95% CI)**				**68**			**34**	**100.0%**	**1.30 [0.76, 1.83]**

Heterogeneity: Tau² = 0.07; Chi² = 3.97, df = 3 (P = 0.27); I² = 24%
Test for overall effect: Z = 4.74 (P < 0.00001)

Std. Mean Difference
IV, Random, 95% CI

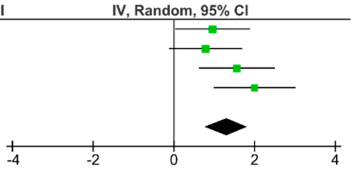

Figure 2S. Meta-Analysis results for corneal graft rejection

Dry eye disease - Adaptive immune system

		DED			Control				Std. Mean Difference
**Study**	**Parameter**	**Mean**	**SD**	**Total**	**Mean**	**SD**	**Total**	**Weight**	**IV, Random, 95% CI**
Bose et al. (2017)	$CD4^+$ Naïve	3	2	16	0.5	0.001	39	3.1%	2.32 [1.58, 3.05]
	$CD4^+$ T_{CM}	14	11	16	2	1	39	3.2%	2.00 [1.30, 2.70]
	$CD4^+$ T_{EM}-T_{RM}	37	20	36	7	4	39	3.3%	2.10 [1.53, 2.67]
	$CD8^+$ Naïve	24	7	16	4.3	2	39	2.7%	4.75 [3.66, 5.84]
	$CD8^+$ T_{CM}	30	10	16	2	3	39	2.8%	4.68 [3.60, 5.76]
	$CD8^+$ T_{EMRA}-T_{RM}	90	10	36	36	18	39	3.1%	3.63 [2.88, 4.38]
Celik (2017)	NLR	2.6	1.2	78	1.84	0.5	60	3.4%	0.79 [0.44, 1.14]
	PLR	138.4	62.6	78	118.5	64.7	60	3.5%	0.31 [-0.03, 0.65]
Ji et al. (2017)	IL17	0.65	0.5	31	0.05	0.001	18	3.2%	1.48 [0.82, 2.13]
	IL22	1.8	0.7	31	0.25	0.15	18	3.1%	2.69 [1.89, 3.50]
Liu et al. (2017)	IL17	1.6	0.2	15	1	0.001	15	2.5%	4.13 [2.80, 5.46]
	IL23	2.05	0.4	15	1	0.001	15	2.6%	3.61 [2.40, 4.83]
	IL6	1.2	0.1	15	1	0.001	15	2.8%	2.75 [1.72, 3.79]
Luo et al. (2017)	IFNγ	25.8	10.6	30	19.3	9.7	30	3.3%	0.63 [0.11, 1.15]
	IL10	56.1	15.3	30	51	11.3	30	3.3%	0.37 [-0.14, 0.89]
	IL17	35.3	17.2	30	39.8	12.6	30	3.3%	-0.29 [-0.80, 0.21]
	IL33	8	3	30	2.5	1.7	30	3.2%	2.23 [1.57, 2.88]
	IL4	4.61	2.37	30	1.54	0.48	30	3.3%	1.77 [1.17, 2.38]
	IL5	32.12	9.36	30	12.38	2.95	30	3.1%	2.81 [2.08, 3.53]
	TGFβ	291	47.6	30	257.9	51.3	30	3.3%	0.66 [0.14, 1.18]
Nicolle et al. (2018)	CCL2	3	2	23	1	0.1	13	3.1%	1.21 [0.47, 1.96]
	CXCL12	2	2.75	32	1	0.2	15	3.2%	0.43 [-0.19, 1.05]
	CXCR4	2.2	1.6	32	1	0.1	12	3.2%	0.86 [0.17, 1.55]
	HLA DR	4	2	37	1	0.5	13	3.1%	1.69 [0.97, 2.41]
	IL6	18	15	30	0.5	0.001	11	3.1%	1.33 [0.57, 2.08]
	CCR2	2.5	2	32	1	0.05	14	3.2%	0.88 [0.22, 1.53]
Tan et al. (2014)	IL17	60	40	20	25	23	20	3.2%	1.05 [0.39, 1.72]
	IL22	11.5	5.5	20	6.5	5	20	3.2%	0.93 [0.28, 1.59]
Yoon et al. (2010)	CXCL10	1.15	0.35	17	1	0.35	15	3.2%	0.42 [-0.29, 1.12]
	CXCL11	1.3	0.7	17	0.8	0.4	15	3.1%	0.84 [0.11, 1.57]
	CXCL9	1.5	0.75	17	0.7	0.5	15	3.1%	1.21 [0.45, 1.97]
	CXCR3	35	8	17	14	10	15	2.9%	2.28 [1.36, 3.19]
**Total (95% CI)**				**903**			**823**	**100.0%**	**1.69 [1.31, 2.07]**

Heterogeneity: Tau² = 1.05; Chi² = 337.62, df = 31 (P < 0.00001); I² = 91%
Test for overall effect: Z = 8.74 (P < 0.00001)

Std. Mean Difference
IV, Random, 95% CI

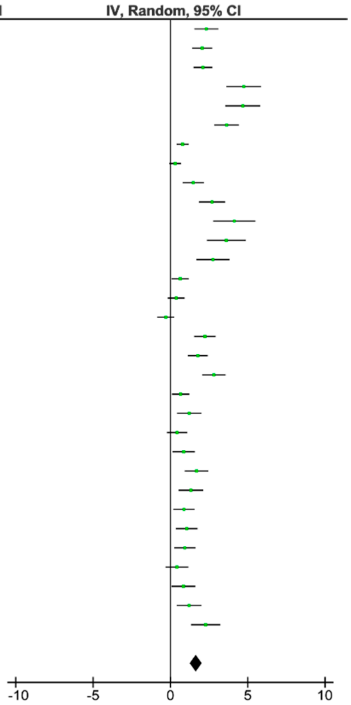

Dry eye disease - Innate immune system

		DED			Control				Std. Mean Difference
**Study**	**Parameter**	**Mean**	**SD**	**Total**	**Mean**	**SD**	**Total**	**Weight**	**IV, Random, 95% CI**
Celik (2017)	NLR	2.6	1.2	78	1.84	0.5	60	10.7%	0.79 [0.44, 1.14]
	PLR	138.4	62.6	78	118.5	64.7	60	10.7%	0.31 [-0.03, 0.65]
Kherirkhah et al. (2015a)	Dendritic cell	111.7	137.3	45	32	24.4	15	10.4%	0.65 [0.06, 1.25]
Kherirkhah et al. (2015b)	Dendritic cell	133.19	25.01	37	19.6	2.8	20	9.4%	5.52 [4.34, 6.70]
Nicolle et al. (2018)	CCL2	3	2	23	1	0.1	13	10.2%	1.21 [0.47, 1.96]
	Dendritic cell	103.5	84.5	25	49.2	43.7	15	10.4%	0.74 [0.07, 1.40]
	IL6	18	15	30	0.5	0.001	11	10.2%	1.33 [0.57, 2.08]
	CCR2	2.5	2	32	1	0.05	14	10.4%	0.88 [0.22, 1.53]
Shetty et al. (2016)	Dendritic cell	52.9	5	52	9.1	1.3	43	8.2%	11.42 [9.71, 13.13]
Tepelus et al. (2017)	Dendritic cell	40	7	12	27	2	7	9.3%	2.16 [0.95, 3.37]
**Total (95% CI)**				**412**			**258**	**100.0%**	**2.27 [1.28, 3.25]**

Heterogeneity: Tau² = 2.33; Chi² = 222.98, df = 9 (P < 0.00001); I² = 96%
Test for overall effect: Z = 4.51 (P < 0.00001)

Std. Mean Difference
IV, Random, 95% CI

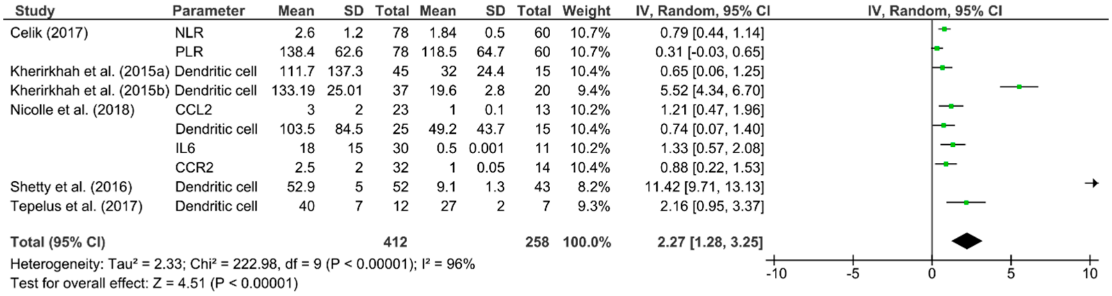

Figure 3S. Meta-analysis for Dry Eye Disease

Glaucoma - Adaptive immune system

Study	Parameter	Glaucoma Mean	Glaucoma SD	Glaucoma Total	Control Mean	Control SD	Control Total	Weight	Std. Mean Difference IV, Random, 95% CI
Gramlich et al. (2013)	IFNγ	18,003	4,390	6	17,189	1,346	9	4.2%	0.26 [-0.78, 1.30]
	IL6	12,073	6,695	6	8,301	2,287	9	4.2%	0.78 [-0.30, 1.87]
	Plasma cells	1.8	1.2	6	0.12	0.14	9	4.0%	2.10 [0.75, 3.46]
	TNFα	907	454	6	538	200	9	4.1%	1.08 [-0.05, 2.20]
Liang et al. (2006)	CCR4	15.8	4.5	18	3.5	0.6	20	4.1%	3.86 [2.74, 4.97]
	CCR5	15.7	5.7	18	2.5	0.6	20	4.2%	3.28 [2.27, 4.28]
	HLA-DR	67.2	5.4	18	23.8	5	20	3.4%	8.18 [6.14, 10.23]
Malvitte et al. (2007)	IFNγ	4,200	900	21	120	80	12	3.8%	5.49 [3.91, 7.07]
	IL10	1,050	600	21	90	40	12	4.3%	1.94 [1.07, 2.81]
	IL12	1,800	400	21	100	100	12	3.9%	5.08 [3.59, 6.56]
	IL13	3,150	900	21	200	100	12	4.1%	3.97 [2.73, 5.21]
	IL17	950	250	21	50	20	12	4.0%	4.36 [3.04, 5.69]
	IL2	10,500	3,500	21	1,000	900	12	4.2%	3.24 [2.14, 4.33]
	IL4	2,000	800	21	200	100	12	4.2%	2.72 [1.72, 3.72]
	IL5	1,200	850	21	80	50	12	4.3%	1.60 [0.78, 2.42]
	IL6	3,062	953	21	1,050	750	12	4.3%	2.21 [1.31, 3.12]
	IL7	1,400	500	21	150	90	12	4.2%	3.01 [1.96, 4.06]
	TNFα	1,200	700	21	70	20	12	4.3%	1.96 [1.09, 2.83]
Taurone et al. (2014)	IL6	85.42	10	7	36	10	4	2.9%	4.52 [1.86, 7.18]
	TGFβ1	81.57	10	7	31.33	10	4	2.9%	4.59 [1.90, 7.29]
	TNFα	60.43	10	7	41	10	4	3.8%	1.78 [0.24, 3.32]
Wong et al. (2015)	IFNγ	0.463	0.081	18	0.206	0.029	18	4.1%	4.13 [2.93, 5.33]
	IL2	0.35	0.063	18	0.181	0.032	18	4.2%	3.31 [2.27, 4.35]
	IL6	0.073	0.039	18	0.248	0.12	18	4.3%	-1.92 [-2.72, -1.11]
	TGFβ	0.319	0.038	18	0.142	0.021	18	3.9%	5.64 [4.11, 7.16]
**Total (95% CI)**				**402**			**312**	**100.0%**	**2.98 [2.21, 3.75]**

Heterogeneity: Tau² = 3.39; Chi² = 266.42, df = 24 (P < 0.00001); I² = 91%
Test for overall effect: Z = 7.59 (P < 0.00001)

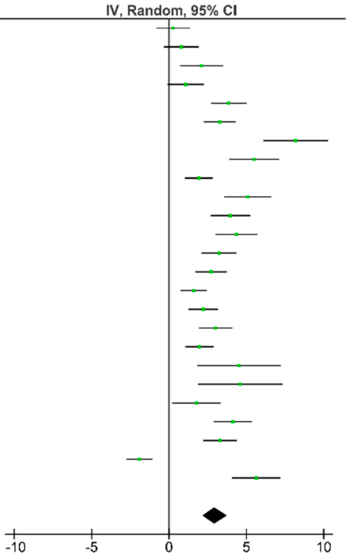

Glaucoma - Innate immune system

Study	Parameter	Glaucoma Mean	Glaucoma SD	Glaucoma Total	Control Mean	Control SD	Control Total	Weight	Std. Mean Difference IV, Random, 95% CI
Baghdasaryan et al. (2018)	Dendritic cells	71.24	61.74	30	34.08	11.7	20	8.1%	0.75 [0.17, 1.34]
Gramlich et al. (2013)	IFNγ	18,003	4,390	6	17,189	1,346	9	7.4%	0.26 [-0.78, 1.30]
	IL1β	15,625	4,194	6	11,262	2,590	9	7.1%	1.24 [0.09, 2.40]
	IL6	12,073	6,695	6	8,301	2,287	9	7.3%	0.78 [-0.30, 1.87]
	IL8	13,394	2,749	6	9,990	1,632	9	7.0%	1.50 [0.29, 2.71]
Malvitte et al. (2007)	IL12	1,800	400	21	100	100	12	6.4%	5.08 [3.59, 6.56]
	IL13	3,150	900	21	200	100	12	6.9%	3.97 [2.73, 5.21]
	IL1β	1,300	1,200	21	90	80	12	7.8%	1.22 [0.45, 2.00]
	IL6	3,062	953	21	1,050	750	12	7.6%	2.21 [1.31, 3.12]
	IL8	4,650	1,383	21	356	93	12	7.0%	3.77 [2.57, 4.97]
Margeta et al. (2018)	CD163+	5.1	0.6	13	2.5	0.3	12	5.8%	5.23 [3.47, 7.00]
Mastropasqua et al. (2016)	Dendritic cells	33.96	18.77	29	7.33	4.96	15	7.9%	1.68 [0.95, 2.40]
Taurone et al. (2014)	IL1β	70.42	10	7	38.33	10	4	5.4%	2.93 [0.97, 4.90]
	IL6	85.42	10	7	36	10	4	4.1%	4.52 [1.86, 7.18]
	TGFβ1	81.57	10	7	31.33	10	4	4.1%	4.59 [1.90, 7.29]
**Total (95% CI)**				**222**			**155**	**100.0%**	**2.43 [1.68, 3.18]**

Heterogeneity: Tau² = 1.70; Chi² = 92.84, df = 14 (P < 0.00001); I² = 85%
Test for overall effect: Z = 6.36 (P < 0.00001)

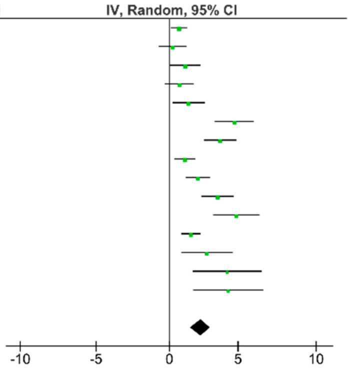

Figure 4S. Meta-analysis for Glaucoma

Graft-vs-host disease - Innate immune system

Study	Parameter	GVHD Mean	GVHD SD	GVHD Total	Control Mean	Control SD	Control Total	Weight	Std. Mean Difference IV, Random, 95% CI
He et al. (2017)	Dendritic cells	119.29	79.78	12	37.57	21.72	10	43.5%	1.29 [0.35, 2.23]
Kheirkhah et al. (2016)	Dendritic cells	148	135	33	122	99	21	56.5%	0.21 [-0.34, 0.76]
**Total (95% CI)**				**45**			**31**	**100.0%**	**0.68 [-0.37, 1.73]**

Heterogeneity: Tau² = 0.43; Chi² = 3.80, df = 1 (P = 0.05); I² = 74%
Test for overall effect: Z = 1.27 (P = 0.20)

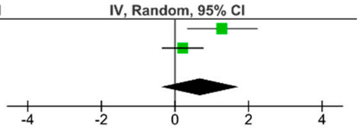

Figure 5S. Meta-analysis for Graft vs Host Disease. Only innate immune system is shown, because no adaptive immune cells were found.

Keratitis - Adaptive immune system

Study	Parameter	Keratitis Mean	Keratitis SD	Keratitis Total	Control Mean	Control SD	Control Total	Weight	Std. Mean Difference IV, Random, 95% CI
		**Bacterial keratitis**							
Yamaguchi et al. (2014)	CCL2	150	60	10	73.7	25.3	14	10.0%	1.71 [0.74, 2.68]
	IL2	5,000	500	10	35,000	1,000	14	0.7%	-34.79 [-45.59, -23.99]
	IL6	7,174	2,430	10	171.8	32.1	14	8.6%	4.35 [2.78, 5.92]
	IL7	80	40	10	70	50	14	10.3%	0.21 [-0.60, 1.02]
	IL10	40	15	10	17.5	4.9	14	9.9%	2.11 [1.07, 3.15]
	IL17a	2,200	250	10	1,350	337	14	9.6%	2.70 [1.53, 3.86]
		**Stromal keratitis**							
Iskeleli et al. (2008)	CD3	71.45	10.47	11	68.64	7.89	10	10.2%	0.29 [-0.57, 1.15]
	CD4	46	9.64	11	43.35	5.53	10	10.2%	0.32 [-0.54, 1.18]
	CD8	31.2	7.34	11	23.23	2.2	10	10.0%	1.38 [0.41, 2.36]
	CD19	10.57	3.2	11	11.16	2.99	10	10.2%	-0.18 [-1.04, 0.68]
	CD20	11.09	3.01	11	10.4	2	10	10.2%	0.26 [-0.60, 1.12]
**Total (95% CI)**				**115**			**134**	**100.0%**	**0.98 [0.01, 1.95]**

Heterogeneity: Tau² = 2.19; Chi² = 93.13, df = 10 (P < 0.00001); I² = 89%
Test for overall effect: Z = 1.99 (P = 0.05)

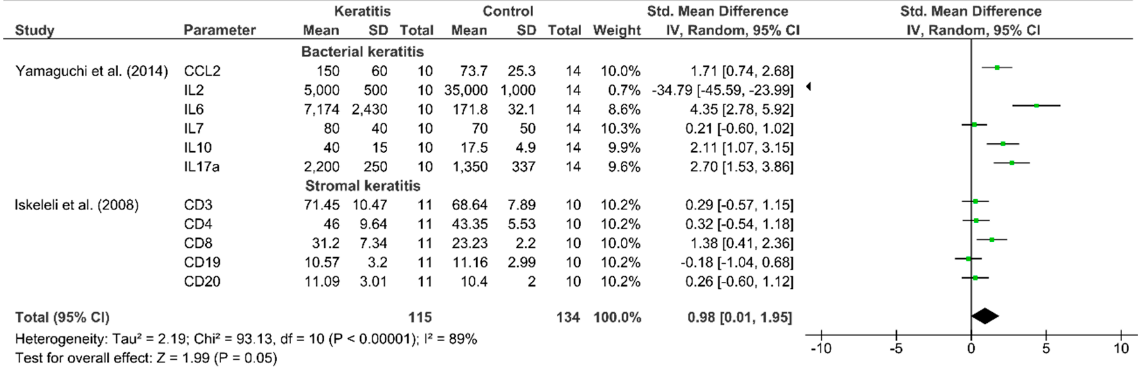

Keratitis - Innate immune system

Study	Parameter	Keratitis Mean	Keratitis SD	Keratitis Total	Control Mean	Control SD	Control Total	Weight	Std. Mean Difference IV, Random, 95% CI
		**Fungal keratitis**							
Cruzat et al. (2011)	Dendritic cells	608.9	812.5	13	49.3	39.6	20	11.1%	1.08 [0.33, 1.83]
		**Bacterial keratitis**							
Cruzat et al. (2011)	Dendritic cells	441.1	320.5	23	49.3	39.6	20	11.2%	1.63 [0.93, 2.33]
Yamaguchi et al. (2014)	Dendritic cells	226.9	37.3	10	22.7	5.9	14	6.4%	8.12 [5.48, 10.76]
	CCL2	150	60	10	73.7	25.3	14	10.6%	1.71 [0.74, 2.68]
	GM-CSF	120	80	10	100	90	14	10.9%	0.22 [-0.59, 1.04]
	IL1β	66.6	26.7	10	13	4	14	10.0%	2.98 [1.75, 4.21]
	IL1Ra	350	170	10	40	30	14	10.2%	2.69 [1.53, 3.86]
	IL6	7,174	2,430	10	171.8	32.1	14	9.1%	4.35 [2.78, 5.92]
	IL8	810	315	10	56.5	33.8	14	9.6%	3.58 [2.21, 4.95]
		**Stromal keratitis**							
Iskeleli et al. (2008)	CD16	16.88	5.75	11	18.33	4.3	10	10.9%	-0.27 [-1.13, 0.59]
**Total (95% CI)**				**117**			**148**	**100.0%**	**2.31 [1.32, 3.30]**

Heterogeneity: Tau² = 2.16; Chi² = 82.34, df = 9 (P < 0.00001); I² = 89%
Test for overall effect: Z = 4.58 (P < 0.00001)

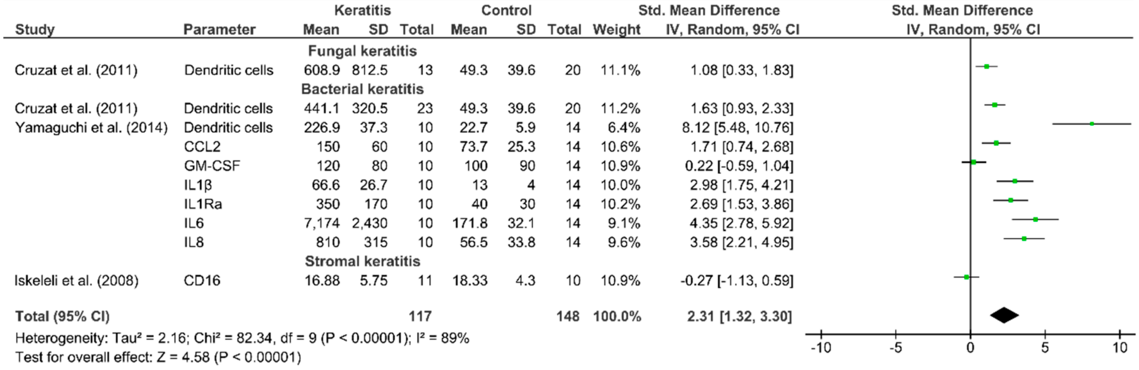

Figure 6S. Meta-analysis for Keratitis

Scleritis - Adaptive immune system

Study	Parameter	Scleritis Mean	Scleritis SD	Scleritis Total	Control Mean	Control SD	Control Total	Weight	Std. Mean Difference IV, Random, 95% CI
Bernauer et al. (1994)	CD20	0.7	0.7	6	0	0.001	12	8.0%	1.70 [0.54, 2.87]
	CD3	32.9	18.7	6	2.4	2.9	12	7.4%	2.71 [1.31, 4.11]
	CD4	17.2	12.2	6	0.8	0.8	12	7.6%	2.28 [0.99, 3.57]
	CD8	12.9	10.4	6	0.9	0.9	12	7.8%	1.95 [0.73, 3.17]
	HLA-DR	65.6	41.1	6	5.1	2.9	12	7.5%	2.49 [1.15, 3.84]
	IL2	2.2	2.1	6	0.1	0.1	12	8.0%	1.70 [0.54, 2.86]
Fong et al. (1991)	CD22	58.56	39.59	9	0.2	0.12	4	7.4%	1.61 [0.22, 3.00]
	CD3	145.7	59.46	9	0.17	0.1	4	6.6%	2.67 [0.97, 4.37]
	CD4	192.37	41.24	9	0.19	0.13	4	4.5%	5.08 [2.46, 7.70]
	CD8	89.96	38.15	9	0.1	0.1	4	6.7%	2.57 [0.90, 4.24]
	HLA-DR	210	59.46	9	1.09	0.06	4	5.6%	3.83 [1.71, 5.95]
Sainz de la Maza et al. (1991)	CD3	84.4	22.35	10	5.41	0.77	13	6.2%	5.20 [3.35, 7.04]
	CD4	139.54	18.43	10	5.76	1.06	13	3.2%	10.66 [7.18, 14.15]
	CD8	65.32	19.03	10	6.76	1.67	13	6.7%	4.51 [2.86, 6.16]
	HLA-DR	45.65	12.03	10	10.43	1.03	13	6.9%	4.29 [2.70, 5.88]
**Total (95% CI)**				**121**			**144**	**100.0%**	**3.16 [2.39, 3.92]**

Heterogeneity: Tau² = 1.55; Chi² = 49.77, df = 14 (P < 0.00001); I² = 72%
Test for overall effect: Z = 8.11 (P < 0.00001)

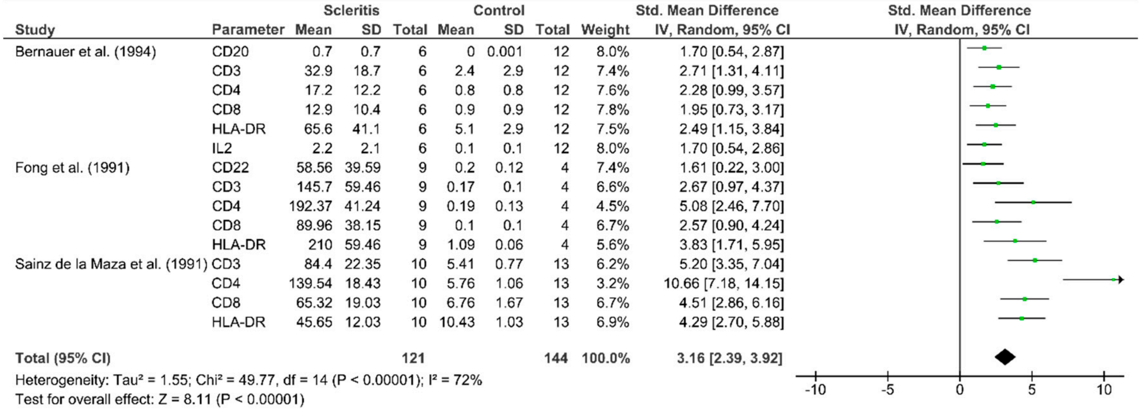

Scleritis - Innate immune system

Study	Parameter	Scleritis Mean	Scleritis SD	Scleritis Total	Control Mean	Control SD	Control Total	Weight	Std. Mean Difference IV, Random, 95% CI
Bernauer et al. (1994)	CD68	26.6	16	6	2.5	2.2	12	14.1%	2.51 [1.16, 3.86]
	HLA-DR	65.6	41.1	6	5.1	2.9	12	14.1%	2.49 [1.15, 3.84]
Fong et al. (1991)	CD1	6.96	5.47	9	0	0.001	4	14.1%	1.39 [0.05, 2.73]
	CD14	43.5	10.91	9	0	0.001	4	9.2%	4.35 [2.03, 6.67]
	CD16	44.56	34.86	9	0	0.001	4	14.1%	1.39 [0.05, 2.74]
	HLA-DR	210	59.46	9	1.46	0.22	4	10.1%	3.83 [1.71, 5.94]
Sainz de la Maza et al. (1991)	CD1	5.04	1.02	10	1.02	0.44	13	11.4%	5.19 [3.35, 7.04]
	HLA-DR	45.65	12.03	10	10.43	1.03	13	12.8%	4.29 [2.70, 5.88]
**Total (95% CI)**				**68**			**66**	**100.0%**	**3.03 [2.05, 4.01]**

Heterogeneity: Tau² = 1.31; Chi² = 21.30, df = 7 (P = 0.003); I² = 67%
Test for overall effect: Z = 6.05 (P < 0.00001)

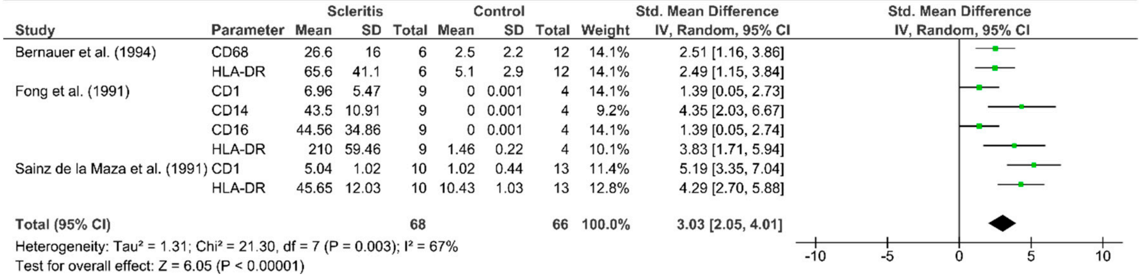

Figure 7S. Meta-analysis for Scleritis

Sjögren's syndrome - Adaptive immune system

Study	Parameter	SS Mean	SS SD	SS Total	Control Mean	Control SD	Control Total	Weight	Std. Mean Difference IV, Random, 95% CI
Fujihara et al. (1999)	CD4	2.1	0.5	5	0.1	0.1	4	2.4%	4.63 [1.49, 7.78]
	CD8	0.75	0.65	5	0.1	0.1	4	4.0%	1.17 [-0.33, 2.66]
Liu et al. (2017)	IL17	2.5	0.3	15	1	0.001	15	3.5%	6.88 [4.88, 8.88]
	IL23	2.1	0.4	15	1	0.001	15	4.2%	3.78 [2.53, 5.04]
	IL6	2.15	0.35	15	1	0.001	15	4.1%	4.52 [3.10, 5.94]
Luo et al. (2017)	IFNγ	22.6	9.1	30	19.3	9.7	30	4.8%	0.35 [-0.16, 0.86]
	IL10	53.3	12.9	30	51	11.3	30	4.8%	0.19 [-0.32, 0.69]
	IL17	41.8	23.1	30	39.8	12.6	30	4.8%	0.11 [-0.40, 0.61]
	IL33	16	1	30	2.5	1.7	30	3.6%	9.55 [7.71, 11.39]
	IL4	8.25	2.77	30	1.54	0.48	30	4.6%	3.33 [2.53, 4.13]
	IL5	53.4	12.2	30	12.38	2.95	30	4.5%	4.56 [3.58, 5.55]
	TGFβ	268.2	54.8	30	257.9	51.3	30	4.8%	0.19 [-0.32, 0.70]
Pflugfelder et al. (2018)	$CD45^+CD11c^+HLA\text{-}DR^+$	292	303.2	9	80.3	63.7	7	4.4%	0.86 [-0.19, 1.90]
	$CD45^+HLA\text{-}DR^+$	357.9	365.7	9	97.6	72.2	7	4.4%	0.88 [-0.17, 1.93]
Tan et al. (2014)	IL17	90	55	20	25	23	20	4.7%	1.51 [0.80, 2.22]
	IL22	16	7	20	6.5	5	20	4.7%	1.53 [0.82, 2.24]
Williams et al. (2016)	B cells	43.2	20	19	18.5	15	21	4.7%	1.38 [0.68, 2.08]
	Lymphocytes	849	323	19	684	148	21	4.7%	0.66 [0.02, 1.29]
	T cells	686	281	19	436	285	21	4.7%	0.87 [0.21, 1.52]
Yoon et al. (2010)	CXCL10	2.5	0.3	16	1	0.35	15	4.1%	4.49 [3.10, 5.88]
	CXCL11	2.3	0.5	16	0.8	0.4	15	4.4%	3.21 [2.11, 4.32]
	CXCL9	2.6	0.6	16	0.7	0.5	15	4.4%	3.34 [2.21, 4.48]
	CXCR3	47	18	16	14	10	15	4.5%	2.19 [1.27, 3.10]
**Total (95% CI)**				**444**			**440**	**100.0%**	**2.42 [1.73, 3.10]**

Heterogeneity: $Tau^2 = 2.46$; $Chi^2 = 340.65$, df = 22 (P < 0.00001); $I^2 = 94\%$
Test for overall effect: Z = 6.93 (P < 0.00001)

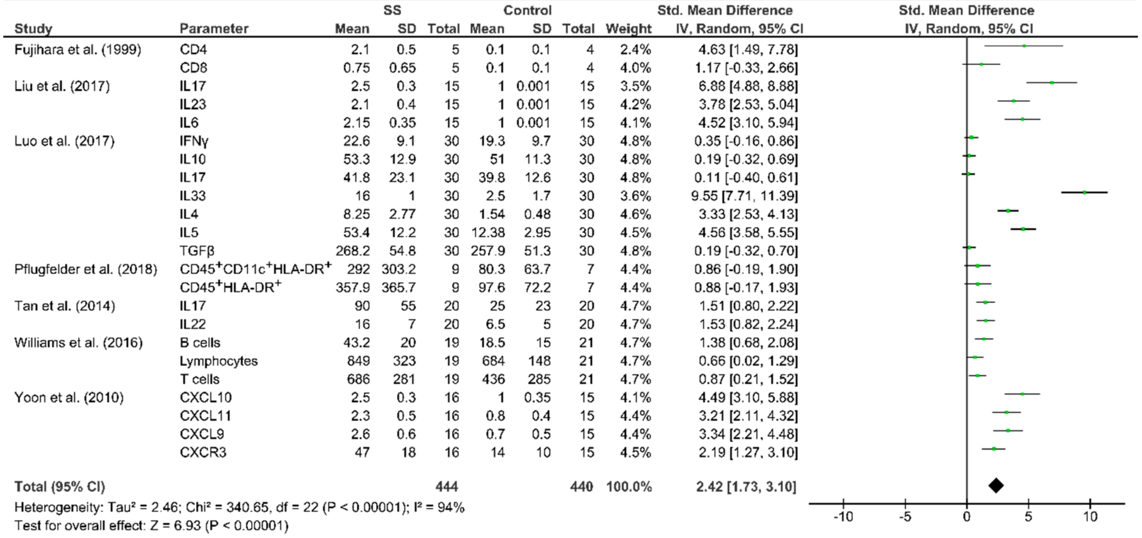

Sjögren's syndrome - Innate immune system

Study	Parameter	SS Mean	SS SD	SS Total	Control Mean	Control SD	Control Total	Weight	Std. Mean Difference IV, Random, 95% CI
Pflugfelder et al. (2018)	$CD45^+CD11c^+CD86^+$	271.6	333.9	8	84.5	63.7	7	25.0%	0.71 [-0.35, 1.76]
Tepelus et al. (2017)	Dendritic cell	71	11	22	27	2	7	23.6%	4.39 [2.90, 5.87]
Williams et al. (2016)	Neutrophils	1.8	0.7	19	5.8	4.5	21	25.8%	-1.19 [-1.87, -0.51]
	NKT cells	43.9	5	19	31.5	6	21	25.6%	2.19 [1.39, 2.99]
**Total (95% CI)**				**68**			**56**	**100.0%**	**1.47 [-0.74, 3.67]**

Heterogeneity: $Tau^2 = 4.78$; $Chi^2 = 66.82$, df = 3 (P < 0.00001); $I^2 = 96\%$
Test for overall effect: Z = 1.30 (P = 0.19)

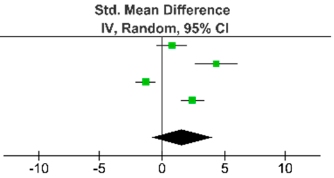

Figure 8S. Meta-analysis for Sjögren’s Syndrome
